# Genome‐Guided Discovery of Vaccine Targets for a Multi‐Epitope Construct Against Multidrug‐Resistant *Enterobacter cloacae*

**DOI:** 10.1002/mbo3.70350

**Published:** 2026-06-24

**Authors:** Maha A. Aljumaa, Fakhria A. Al‐Joufi, Ghulam Nabi, Mengue Ngadena Yolande Sandrine

**Affiliations:** ^1^ Department of Biology, College of Science Princess Nourah bint Abdulrahman University Riyadh Saudi Arabia; ^2^ Department of Pharmacology, College of Pharmacy Jouf University Aljouf Saudi Arabia; ^3^ Department of Zoology The Universiyt of Swat Khyber Pakhtunkhwa Swat Pakistan; ^4^ Department of Animal Biology and Physiology, Laboratory of Animal Physiology, Faculty of Science University of Yaoundé 1 Yaoundé Cameroon

**Keywords:** *Enterobacter cloacae*, epitope, multidrug resistant, OmpA protein, vaccine design

## Abstract

Multidrug‐resistant (MDR) *Enterobacter cloacae* has emerged as a serious public health threat, particularly in hospital settings, due to its ability to cause severe infections and rapidly acquire antibiotic resistance. The limited effectiveness of current antibiotics highlights the urgent need for alternative preventive strategies such as vaccination. In this study an immunoinformatics‐based approach was employed to design a multi epitope vaccine (MEV) targeting the conserved and surface exposed outer membrane protein A (OmpA) of MDR *E. cloacae*. The entire proteome was screened to identify suitable vaccine targets based on antigenicity, allergenicity and homology analysis. Highly immunogenic B‐cell, MHC class I and MHC class II epitopes were predicted and selected for vaccine construction. The selected epitopes were linked using suitable linkers, while the 50S ribosomal L7/L12 protein was incorporated as an adjuvant. The designed MEV construct was evaluated through structural modelling, molecular docking with TLR4 receptor, immune simulation, population coverage analysis, codon optimization and in silico cloning. The vaccine candidate demonstrated strong antigenicity, non‐allergenicity, structural stability, and favourable physicochemical properties. Population coverage analysis indicated 99.92% global coverage. Molecular docking revealed stable and energetically favourable interactions with TLR4, while immune simulation predicted strong humoral and cellular immune responses, including memory cell formation and a Th1‐biased cytokine profile. Codon optimization indicated favorable expression potential in an *E. coli*. Overall, the proposed MEV predicted a promising, safe, stable and broadly protective vaccine candidate against MDR *Enterobacter cloacae*.

## Graphical abstract

Multidrug‐resistance (MDR) *Enterobacter cloacae* was targeted using a genome wide immunoinformatics approach. Conserved and surface exposed outer membrane protien A (OmpA) was identified as the prime vaccine target following proteome screening, antigenicity, allergenicity, and homology analysis. Highly immunogenic B‐Cell, MHC class I and MHC class II epitopes were selected and assembled into a multi epitope vaccine construct using suitable linkers and the 50S ribosomal L7/L12 protien as an adjuvant. Structural modeling, molecular docking with TLR4, immune simulation and population coverage analysis demonstrated strong antigenicity, structural stability, favourable receptor interactions and broad global coverage (99.92%). Codon optimization and in silico cloning confirmed high expression potential in *E. coli*. The designed vaccine construct represents a promising, safe and broadly protective candidate against MDR *Enterobacter cloacae*.

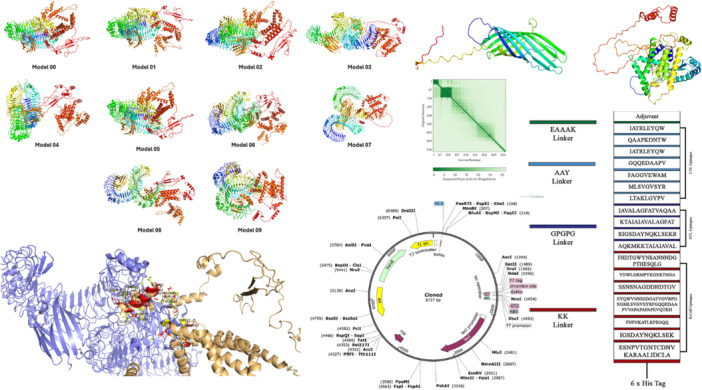

## 1 Introduction

Multidrug‐resistant (MDR) *Enterobacter cloacae* has emerged as a major concern in the modern healthcare system worldwide. It is an important Gram‐negative oppurtunistic pathogen associated with hospital‐acquired infections, such as septicemia, pneumonia, urinary tract infections, and surgical site infections. It exhibits remarkable genetic flexibility, allowing resistance determinants to be acquired and spread by plasmids, transposons, and integrons. The high incidence of extended‐spectrum β‐lactamase‐producing and carbapenemase‐producing *E. colacae* strains have significantly reduced the effectiveness of last‐line antibiotics (Mezzatesta et al. 2012). This has led to increased therapeutic failures and mortality as a result of infection. MDR E. *cloacae* has a high burden in immunocompromised patients and intensive care units. Traditional antimicrobial agents are gradually becoming less effective against the resistant strains. Therefor, there is a critical need to develop alternative and sustainable strategies to reduce MDR E. *cloacae* infections. Preventive interventions, particularly immunization, offer a feasible response to this increasing public health threat (John et al. 1982).

The current strategies for treating E. *cloacae* infections rely heavily on antibiotic therapy and strict infection control. However, antibiotic‐based treatment induces selective pressure, which accelerates resistance development and spread. Moreover, the formation of biofilms and the change in membrane permeability in resistant strains reduce the effectiveness of most antibiotics (Paauw et al. 2008). Vaccination has emerged as a promising alternative strategy to standard medicines in this situation. The vaccines, instead of antibiotics, are preventive in nature, which implies that they trigger the host's immune system to generate an immune‐mediated response to infections just before infections can spread. They also reduce antibiotic use, so indirectly limiting the development of resistance. Furthermore, vaccines provide long‐term protection and contribute to herd immunity (Subedi et al. 2025). Unlike broad spectrum antibiotics, vaccines are less likely to disrupt the commensal microbiota and may reduce antibiotic driven selection pressure. Thus, an effective *E. cloacae* vaccine‐based therapies offer a long‐term and cost‐effective approach to controlling MDR bacterial infections. The *cloacae* vaccination would significantly reduce the disease's prevalence and healthcare costs (Lee et al. 2025).

Recent developments in immunoinformatics and genome‐based vaccine design have turned the traditional field of vaccinology into an efficient and rational computational science. Such methods allow screening whole genomes of pathogens systematically, thereby discovering conserved, antigenic, non‐allergenic, non‐toxic, and non‐homologous epitopes. Through the use of a combination of epitope prediction algorithms, the researchers can select epitopes of the T‐cell and B‐cell that will effectively produce a humoral and cellular immune response (Fu et al. 2025). The genome‐based approaches also reduce the risk of antigenic variability and immune escape. Computational design saves a significant amount of time, money, and experimental complexity as compared to traditional laboratory procedures. Furthermore, immunoinformatics tools can quickly calculate antigenicity, allergenicity, and population coverage without requiring extensive wet‐lab experimentation at the initital screening stage. Multi‐epitope vaccines created *in silico* have the ability to contain a variety of immunogenic areas. These vaccinations have higher specificity and immunogenicity than single‐antigen vaccines. As a result, immunoinformatics‐based approaches are most suited to attacking complicated MDR pathogens such as *E. cloacae* (Fu et al. 2025).

The vaccine target was outer membrane protein A (OmpA) because it is essential in bacterial virulence and survival. OmpA is a highly conserved surface protein, which is central in host cell adhesion, invasion, immune evasion, and biofilm formation (Krishnan and Prasadarao 2012; Limongi et al. 2026). Its conservation in a variety of Enterobacter strains predisposes it as a good candidate for the development of a broad‐spectrum vaccine. Its surface accessibility increases host cell recognition, as well. Previous studies have shown that OmpA is capable of eliciting intense immunological reactions in a range of Gram‐negative bacteria (Naveed et al. 2022). The development of a multi‐epitope vaccine generated through the design of OmpA allows the incorporation of multiple antigen regions, which contribute to the immune responses. The probability of a single epitope mutation as a means of escaping immunity is also minimized by this strategy. Population coverage testing is done to ascertain that the epitopes chosen will bind to the different alleles of the human leukocyte antigen. Furthermore, proper adjuvants may also be included to stimulate the innate immune system and increase the efficacy of vaccines; thus, all these factors justify the choice of OmpA as the best vaccine target (Hossain et al. 2024).

The current study includes structural modeling, molecular docking, and immune simulation analyses as fundamental elements of current computational vaccine development. Structural modeling gives an understanding of the three‐dimensional structure, stability, and folding properties of the vaccine construct of interest. The analysis of molecular docking enables evaluating the interaction between the vaccine and immune receptors, including Toll‐like receptors that play a pivotal role in the formation of innate immunity reactions. The magnitude, duration and pattern of antibody‐ and T‐cell‐ mediated immune responses after immunization are also predicted by immune simulations. These analyses help predict the immunogenic potential of the vaccine construct before experimental validation. The combination of these methods reduces experimental failure rates and improves vaccine optimization.

Thus, the current study aims to build an anti‐*Enterobacter cloacae* multi‐epitope vaccine against multidrug‐resistant *Enterobacter cloacae* using advanced Immunoinformatics techniques. The study focuses on OmpA as an antigen of interest and evaluates the construct using population coverage, structural modeling, molecular docking, and immunological simulations. This technique seeks to provide a logical foundation for the development of an effective and broadly protective vaccination candidate.

## 2 Materials and Methods

### 2.1 Protein Target Selection AND Profiling

The proteome was screened and the amino acid sequence of Outer membrane protein A (OmpA) from *Enterobacter cloacae* subsp. *Cloacae* was retreived from the UniProtKB database (https://www.uniprot.org) under the UniProt ID A0A0H3CK16 (Consortium 2019). To examine the immunogenic potential, the protein was submitted to the server VaxiJen v2.0 (https://www.ddg-pharmfac.net/vaxijen/VaxiJen/VaxiJen.html) (Doytchinova and Flower 2007). Using the bacterial model with the threshold set to 0.4, the sequence generated an antigenicity score of 0.7407, thus characterizing it as a probable antigen. Furthermore, the allergenic potential of the candidate was assessed using AllerTOP v2.1 (https://www.ddg-pharmfac.net/allertop_test), which demonstrated the likelihood of the protein being a non‐allergen. Finally, homology with the host proteome was undertaken to reduce any chances of autoimmunity using the BLASTp algorithm (https://blast.ncbi.nlm.nih.gov/Blast.cgi?PAGE=Proteins) against the Homo sapiens proteome to confirm that no significant similarity existed in human proteins (Dimitrov et al. 2014; Stover and Cavalcanti 2017).

### 2.2 Prediction of the Secondary Structure

The secondary structure elements of the OmpA protein were analyzed using the PSIPRED 4.0 server (https://bioinf.cs.ucl.ac.uk/psipred). This software employs a two‐level neural network, which was constructed utilizing Position‐Specific Scoring Matrices generated through PSI‐BLAST. From the results, it was possible to identify the locations of alpha‐helix, beta‐strand, and coil sections, which helped in understanding the stability as well as the accessible portions as epitopes (McGuffin et al. 2000).

### 2.3 Prediction of the Tertiary Structure

For the prediction of tertiary structure, AlphaFold 3 through AlphaFold Server (https://alphafold.ebi.ac.uk) was utilized to predict the preferred OmpA vaccine target. Upon entering the amino acid sequence, the diffusion model produced accurate structural predictions. To check the quality, the predicted Local Distance Difference Test score (pLDDT) was used, which provides information about local confidence on an index score between 0 and 100. Additionally, we checked Predicted Aligned Error (PAE) to verify the precision in domain packing and global structure (Nussinov et al. 2022).

### 2.4 B‐Cell Linear Epitopes Prediction

Linear B‐cell epitopes were predicted using the IEDB analysis resource portal (https://www.iedb.org). Continuous epitopes were predicted by the algorithm BepiPred‐2.0, operating at a default threshold of 0.5. This method uses a random forest algorithm that is trained on epitopes annotated from antibody‐antigen protein structures. These predicted epitopes were further analyzed for their surface accessibility and antigenicity to ensure their interaction with B‐cell receptors and their ability to induce a humoral immune response (Vita et al. 2025).

### 2.5 MHC Class‐I Epitopes Prediction

The binding of CTL epitopes to MHC Class‐I alleles was predicted by using the IEDB T‐cell epitope prediction tool (https://tools.iedb.org/mhci). Predictions were made through the Artificial Neural Network ANN 4.0 method. A reference set of frequent HLA alleles was selected to ensure broad population coverage. Predicted epitopes were ranked based on their IC50 values (inhibitory concentration 50)‐a peptide showing an IC50 < 20 nM was classified as a high‐affinity binder. The percentile rank was also used as a filter for candidates; a low percentile rank shows higher binding affinity (Roomp et al. 2010).

### 2.6 MHC Class‐II Epitopes Prediction

HTL epitopes restricted to MHC Class‐II alleles were predicted using the IEDB consensus method, version 2.22 (https://tools.iedb.org/mhcii). This integrated approach combines the predictions of NN‐align, SMM‐align, and Sturniolo to give a more robust prediction. The prediction was done against a reference set of 27 human HLA‐DR, HLA‐DQ, and HLA‐DP alleles. All epitopes were evaluated based on percentile rank and adjusted rank, with both values lower meaning better binding. The top‐scoring peptides were further assessed for their ability to induce a CD4 + T‐cell response (Andreatta et al. 2018).

### 2.7 Construction of the Vaccine

The final vaccine construct combined the highest‐priority MHC‐I, MHC‐II, and B‐cell epitopes into one multi‐epitope design. An adjuvant consisting of the 50S ribosomal protein L7/L12 was added to its N‐terminal to enhance its immunogenicity. Specific linkers were selected to provide the proper independent folding and presentation of each domain. The adjuvant is connected via an EAAAK rigid linker to the first epitope. AAY linkers separate MHC‐I epitopes in order to support proteasomal processing, while MHC‐II epitopes are joined with sequences of GPGPG. KK sequences link the B‐cell epitopes, which help in the maintenance of their conformational integrity. At the C‐terminus, a 6xHis tag is placed to help in future purification and identification (Naveed et al. 2021).

### 2.8 Vaccine Antigenicity and Allergenecity Assessment

The antigenicity of the final multi‐epitope vaccine construct was appraised by VaxiJen v2.0 (https://www.ddg-pharmfac.net/vaxijen/VaxiJen/VaxiJen.html) (Doytchinova and Flower 2007). It carries out the analysis with the bacterial model on an amino acid sequence using the threshold of 0.4 as a default. Furthermore, the allergenic potential of the recombinant protein has been assessed by using AllerTOP v2.1 (https://www.ddg-pharmfac.net/allertop_test), confirming that the construct is non‐allergenic and safe to use (Dimitrov et al. 2014).

### 2.9 Vaccine Physicochemical and Solubility Analysis

The ExPASy ProtParam tool (https://web.expasy.org/protparam) was used to evaluate the physicochemical properties of the multi‐epitope vaccine construct. Parameters such as molecular weight (MW), theoretical pI, instability index (II), aliphatic index (AI), and GRAVY have been used to determine the levels of stability and hydrophilicity of the protein (Azimi et al. 2024). In addition, the potential of this candidate was assessed to express solubility in E. coli, with the use of SoluProt (https://loschmidt.chemi.muni.cz/soluprot). This machine learning predictor is based upon various features within the sequence to predict the solubility of the protein when over‐expressed, giving it a solubility score that helps determine manufacturability (Hon et al. 2021).

### 2.10 IEDB Population Coverage Analysis

The estimate of the range over which the prioritized T‐cell epitopes would be useful worldwide was done using the Population Coverage Tool from the IEDB database (https://tools.iedb.org/population). The predicted MHC Class I and Class II epitopes, together with the HLA alleles they bind to, were used to provide the probability of coverage based on the whole global population. This would give an estimate of the proportion of people who could potentially respond to the multi‐epitope vaccine. This is based on the variability of the human MHC alleles in different regions (Misra et al. 2011).

### 2.11 Structure Modeling of the Vaccine Construct

The final vaccine secondary structure was analyzed using the PSIPRED 4.0 tool (https://bioinf.cs.ucl.ac.uk/psipred). The software predicts the structure by creating an analysis profile through the use of two feed‐forward neural networks that make predictions based on the results from the PSI‐BLAST search. The results allowed us to examine what percentage of the protein consists of alpha helices, beta strands, and random coils, and where these are present in order to interpret the stability and conformation of the chimeric protein (Misra et al. 2011).

The 3D structure of the designed multi‐epitope vaccine construct was predicted using the AlphaFold AI algorithm (https://alphafold.ebi.ac.uk). The entire AA sequence was submitted as input data for structuring. For ascertaining the level of confidence in the predictions, the values predicted were checked using the local distance difference test (pLDDT) for local confidence, and the predicted aligned error (PAE) matrix that evaluates how well differing domains might interlock and align with each other. On the basis of this analysis, the best model having the highest confidence levels with good structural integrity was selected for analysis and validation (Jumper et al. 2021).

### 2.12 Molecular Docking With Human TLR4 Receptor

For examining the interaction of the designed vaccine with the host innate immune system, we conducted a molecular docking analysis using the ClusPro 2.0 software. In this study, we utilized the human Toll‐like receptor 4 protein structure with the bound MD‐2 protein, as obtained from the Protein Data Bank (PDB ID: 4G8A) (https://www.rcsb.org). Before the completion of the docking analysis, the receptor structure had to be pre‐processed by the removal of water and covalently bound compounds. In this approach, the vaccine construct is considered the ligand, and the other molecule, TLR4‐MD2, is the receptor. This process involves rigid body docking using the PIPER engine in the ClusPro server (https://cluspro.org/help.php), and the docking process involves the use of the FFT correlation approach to yield thousands of potential structures. These structures can then be clustered based on the RMSD value, and the complex with the the top‐ranked cluster with favorable ClusPro/PIPER energy scores (Bank 1971; Kozakov et al. 2017).

### 2.13 Interaction Profiling

The complex formation between the docked vaccine molecule and the TLR4 receptor was investigated and demonstrated using the PyMOL software (https://www.pymol.org). By concentrating on the top‐ranked docked complex molecule, the key residues involved in the complex formation were identified. The particular residues responsible for the intermolecular forces, such as hydrogen bonding and hydrophobic interactions, were identified to clarify the formation of the complex molecule. The bond length was measured, and the amino acid residues involved in the complex molecule were highlighted (DeLano 2002).

### 2.14 Normal Mode Analysis of the Docked Complex

The docked complex of the receptor and the vaccine was analyzed on the iMODS web server (http://imods.chaconlab.org) to analyze how the complex holds together and how it moves. The iMODS web server relies on Normal Mode Analysis in internal coordinates to determine whether the system is flexible or stable. The deformability extent, as well as the determination of B‐factors, helped in understanding how residues deform, as well as how atoms move, respectively. To quantitatively analyze the stiffness of the motion, the eigenvalue was analyzed, as the higher the eigenvalue, the stiffer the motion, as it would take more force to deform the system. Then the covariance matrix was generated to identify the correlated, uncorrelated, and anti‐correlated motions among the residues. Lastly, to analyze the elastic network model to identify the atomic pairs connected by springs, hence determining rigid and flexible parts of the protein (López‐Blanco et al. 2014).

### 2.15 Immune Simulations

The C‐ImmSim server (https://kraken.iac.rm.cnr.it/C-IMMSIM/index.php) was employed to delineate how the multi‐epitope vaccine would interact with the immune system. This agent‐based model simulated a typical vaccination schedule by delivering three doses at set intervals, hence representing a prime‐boost immunization at timeline steps 1, 84, and 168 (weeks 0, 4, and 8). A total run of 1050 time steps was considered. From the output of the C‐ImmSim simulation, the increases of IgG and IgM were measured, along with tracking several immune cell populations like B‐cells, Th, and Tc. Besides these parameters, cytokine responses were analysed, focusing on IFN‐γ and IL‐2, to confirm the realization of a strong and durable adaptive immune response (Laubenbacher et al. 2024; Rapin et al. 2010; Stolfi et al. 2022).

### 2.16 Codon Optimization and In Silico Cloning

The amino acid sequence was reverse‐translated into a DNA sequence with the Reverse Translate tool (https://www.bioinformatics.org/sms2/rev_trans.html) to make sure that the vaccine would reach the host system efficiently. Then, we used the Java Codon Adaptation Tool (JCAT) (https://www.jcat.de) to optimize the codons for Escherichia coli strain K12 preferences. CAI and the percentage of GC content were checked to ensure good quality of optimization, minimizing transcriptional mismatches and allowing for stable mRNA (Grote et al. 2005; Stothard 2000).

After optimization, *in silico* cloning was done by using SnapGene (https://www.snapgene.com) to construct the recombinant plasmid. The optimized gene was inserted into appropriate restriction sites‐ BamHI and HindIII, for example, in the multiple cloning site of the pET‐28(+). The final construct was visualized to confirm that the insert is in the correct orientation and the open reading frame integrity relative to the vector's promoter and terminator regions (Al‐Kanany and Othman 2020).

## 3 Results

### 3.1 Identification and Selection of Target Protein

The whole proteome of *Enterobacter cloacae* was screened in order to identify potential vaccine candidate proteins. After considering surface accessibility and protein essentiality, Outer membrane protein A (OmpA; UniProt Accession: A0A0H3CK16) was selected for further analysis. VaxiJen v2.0 was then used to assess the immunogenic potential of this protein, classifying OmpA as a probable antigen with a score of 0.7407 using a 0.4 threshold. Allergenecity assessment was performed with AllerTOP v2.1, classifying the protein as a probable non‐allergen. A homology search against the proteome of Homo sapiens showed no meaningful similarity to host proteins, which reduces the chances of autoimmune responses.

### 3.2 Structural Analysis of Target Protein

**Figure 1:**
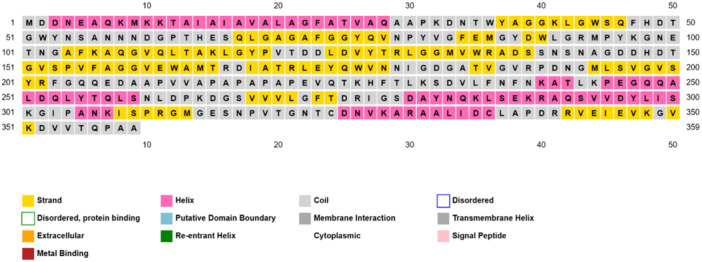
Graphical representation of the secondary structure prediction generated by PSIPRED. The annotation grid maps the distribution of structural elements along the amino acid sequence, where pink blocks indicate alpha‐helices, yellow blocks represent beta‐strands, and grey blocks denote random coils.

The secondary structure for the OmpA vaccine target, described according to the PSIPRED prediction, indicated a mixed secondary structure composed of β‐strands, α‐helices, and coil regions. The N‐terminal, spanning residues 40 to 170, has beta‐strands predominantly, indicating the presence of a transmembrane beta‐barrel structure (Figure 1). In contrast, the C‐terminal, spanning 230 to 359, is highly helical and exists apart from the N‐terminal domain, which has a large coil region dominated by random coils.

**Figure 2:**
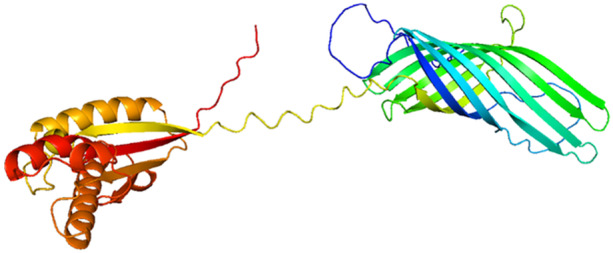
Three‐dimensional structure of the OmpA vaccine target predicted by AlphaFold. The model illustrates the protein's bi‐modular topology, featuring an N‐terminal beta‐barrel domain (blue/green) connected to a C‐terminal alpha‐helical globular domain (orange/red). The rainbow coloring represents the N‐to‐C terminal progression.

The AlphaFold‐predicted tertiary structure supported a two‐domain organization, typical of outer membrane proteins. The three‐dimensional structure displays a distinct beta‐barrel domain in the N‐terminus (blue and green portions) designed to cross the cell membrane. The C‐terminal domain (orange and red portions) with mostly alpha‐helical structure likely corresponds to the periplasmic area. The folding strategy indicates the presence of a stable protein with the capacity to be epitope‐mapped (Figure 2).

### 3.3 Prediction and Selection of B‐Cell and T‐Cell Epitopes

**Figure 3:**
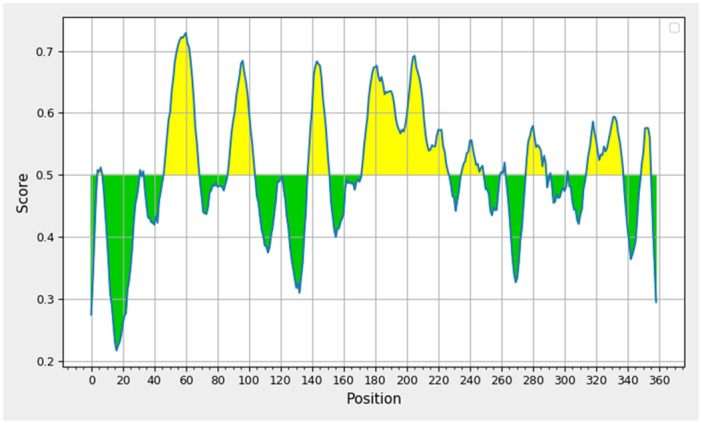
Scoring matrix of the predicted B‐cell epitopes.

The IEDB analysis identified specific epitopes that are potentially involved in antibody recognition. In the antibody response, seven linear B‐cell epitopes were proposed to be involved in humoral immunity using BepiPred‐2.0 software. These peptides have lengths ranging from 13 to 55 amino acids, an average antigenicity score of 0.505, and an epitope score of 0.729. The top epitope ranges from amino acids 173 to 227, consisting of 55 amino acids. Table 1 below represents the predicted B‐cell epitopes, and Figure 3 below represents the scoring parameters for the predicted epitopes.

**Table 1:** Predicted B‐cell epitopes.

No.	Start	End	Peptide sequence	Length (aa)
1	47	69	FHDTGWYNSANNNDGPTHESQLG	23
2	88	104	YDWLGRMPYKGNETNGA	17
3	139	152	SSNSNAGDDHDTGV	14
4	173	227	EYQWVNNIGDGATVGVRPDNGMLSVGVSYRFGQQEDAAPVVAPAPAPAPEVQTKH	55
5	236	249	FNFNKATLKPEGQQ	14
6	277	289	IGSDAYNQKLSEK	13
7	315	338	ESNPVTGNTCDNVKARAALIDCLA	24

For the induction of efficient cellular immunity, high‐affinity T‐cell epitopes were screened. For MHC Class‐I binding peptides, seven peptides were picked because of their low IC50 values (< 20 nM) that ensure good interactions with human leukocyte antigens. For the epitope “IATRLEYQW,” the highest binding affinity is observed with HLA‐B*58:01 (IC50 = 3.34 nM) and also with HLA‐B*57:01 alleles. Table 2 represents the predicted and selected MHC class‐I epitopes based on IC50 values < 20 nM.

**Table 2:** Selected MHC class‐I epitopes.

Peptide sequence	Start	End	Length(aa)	HLA allele	Median percentile rank	SMM IC_50_ (nM)
IATRLEYQW	48	56	9	HLA‐B*58:01	0.20	3.34
QAAPKDNTW	28	36	9	HLA‐B*58:01	0.20	10.19
IATRLEYQW	48	56	9	HLA‐B*57:01	0.20	13.03
GQQEDAAPV	24	32	9	HLA‐A*02:06	0.50	13.69
FAGGVEWAM	36	44	9	HLA‐B*35:01	0.30	13.98
MLSVGVSYR	14	22	9	HLA‐A*68:01	0.30	14.74
LTAKLGYPV	52	60	9	HLA‐A*68:02	0.30	18.14

Moreover, four MHC class II epitopes have been prioritized with the objective of activating CD4 + T‐helper cell responses. The IC50 values of all shortlisted epitopes are less than 20 nM, signifying very good binding affinity. The epitope “IAVALAGFATVAQAA” demonstrated the highest binding affinity (IC50 < 20 nM) with the allele “HLA‐DQA1*05:01/DQB1*03:01.” Table 3 below represents the selected MHC Class‐II epitopes.

**Table 3:** Selected MHC class‐II epitopes for the construction of vaccine chimera.

Peptide sequence	Start	End	Length (aa)	HLA Allele	Median percentile rank	SMM IC_50_ (nM)
IAVALAGFATVAQAA	16	30	15	HLA‐DQA1*05:01/DQB1*03:01	0.73	13
KTAIAIAVALAGFAT	11	25	15	HLA‐DQA1*05:01/DQB1*03:01	0.94	15
RIGSDAYNQKLSEKK	36	50	15	HLA‐DRB5*01:01	0.43	16
AQKMKKTAIAIAVAL	6	20	15	HLA‐DQA1*05:01/DQB1*03:01	0.05	18

### 3.4 Design of Multi‐Epitope Vaccine Construct

**Figure 4:**
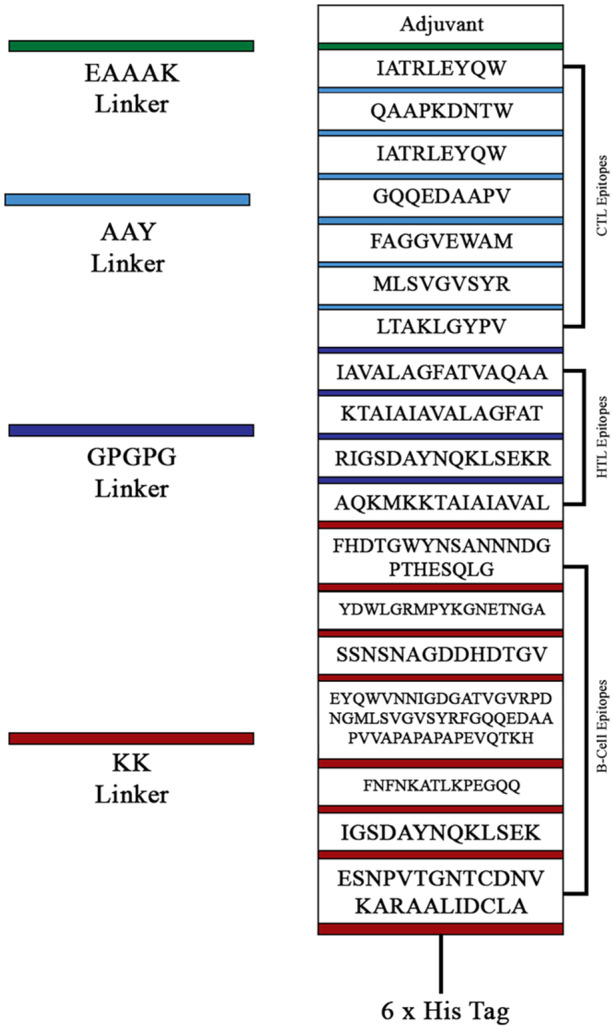
The structure of the vaccine chimeric construct, indicating the adjuvant, linkers, epitopes, and histidine tag.

The final multi‐epitope vaccine was constructed by concatenating the most antigenic fragments that were identified throughout the screening process. To enhance the strength of the immune response, the 50S ribosomal L7/L12 protein from the bacterium Thermus thermophilus was incorporated as an adjuvant at the N‐terminus of the vaccine molecule. A stiff linker EAAAK ensured that the adjuvant was properly distanced from the initial epitope, facilitating appropriate conformational folding with well‐defined limits between domains. In this case, the vaccine is made up of seven MHC Class‐I epitopes, four MHC Class‐II epitopes, and seven linear B‐cell epitopes spread by particular linkers AAY, GPGPG, and KK, which assist with recognition by the proteasome, flexibility necessary for the response by helper T‐cells, and maintenance of conformational epitopes necessary for recognition by B‐cells, with a 6xHis‐tag at the C‐terminus facilitating purification. The chimeric protein established by this vaccine has 476 amino acids. Figure 4 below represents the structure of the vaccine construct. The vaccine construct prioritizes highly promiscuous epitopes—notably IATRLEYQW and QAAPKDNTW for MHC Class‐I, and IAVALAGFATVAQAA for MHC Class‐II—which demonstrated exceptional binding affinities (low IC_50_ values) across a broad range of HLA alleles to improve predicted HLA coverage across diverse populations.

The physicochemical characteristics of the vaccine were also analysed to determine its potential for stability and ease of processing. Using ExPASy ProtParam, results indicated that the molecular weight of the vaccine was 50.1 kDa, and the theoretical pI was 7.68, suggesting a slightly basic protein molecule. With an instability index of 21.48, the vaccine was stable, as the index was below 40. The aliphatic index of 75.04 suggested relatively good thermostability, and the GRAVY index of −0.326 indicated the vaccine to be hydrophilic, with preferences towards aqueous interaction. Solubility probability, as determined from SoluProt, was 0.537, suggesting moderate predicted solubility upon expression in *E. coli*.

### 3.5 Antigenicity, Allergenicity, and Toxicity Evaluation

VaxiJen v2.0 was employed for a quantitative check of how well the designed multi‐epitope vaccine might trigger the immune response. A run using the bacterial model with a threshold of 0.4 resulted in a score for the vaccine of 0.7364, indicating it is likely to be an antigen suggesting that the construct may possess antigenic potential. The prediction came back as a probable non‐allergen, pointing to a favourable safety profile with a low risk of allergic responses at the host level.

### 3.6 Population Coverage Analysis

**Figure 5:**
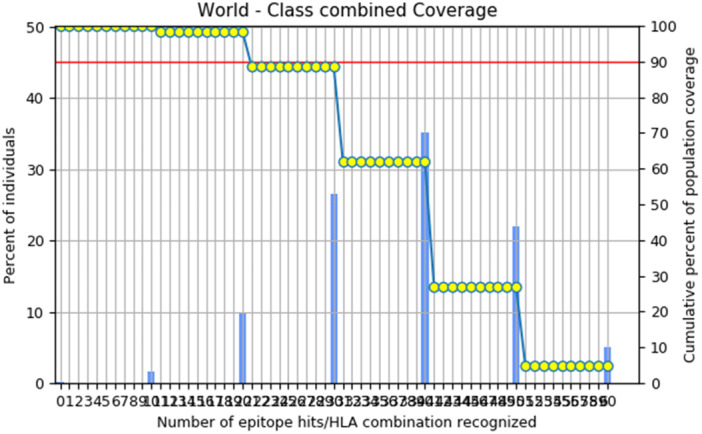
Global population coverage predicted by IEDB.

The efficacy of a multi‐epitope vaccine depends largely on its effectiveness in accommodating a wide range of HLA alleles. Using the database in the IEDB resource named population coverage, the effectiveness in a wide range of the globe in terms of the prioritized MHC Class I and II epitopes was determined. The selected epitopes showed a predicted global population coverage of 99.92%. The data also indicates a high degree of epitope HLA pair interactions, with a hit score averaging 38.07, pointing towards a large number of interactions in every individual. PC90 of 28.52 indicates the minimum number of epitope‐HLA combinations predicted to be recognized by 90% of the population. Figure 5 below represents the global population coverage predicted by IEDB.

### 3.7 Vaccine Structure Modelling and Validation

**Figure 6:**
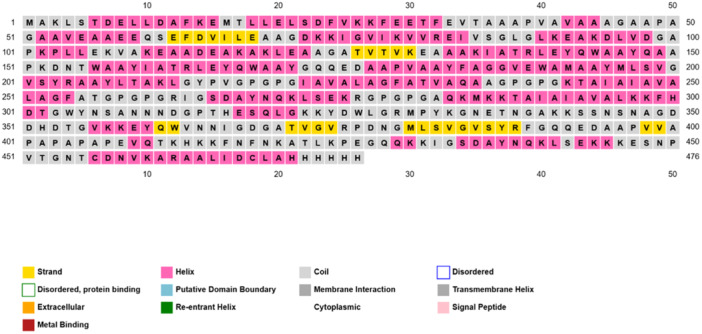
The secondary structure of the designed vaccine construct predicted by PsiPred, indicating Helix, Coils, and Beta Sheets.

The secondary structure prediction performed by PSIPRED correlates well with the 3D structure, with areas of alpha helices, beta strands, and random coils scattered throughout the fusion protein. The predominance of the random coils is largely due to the pliable GPGPG and KK linking segments that connect the epitopes, which are essential for proper antigen presentation. Figure 6 represents the secondary structure of the designed vaccine construct.

**Figure 7:**
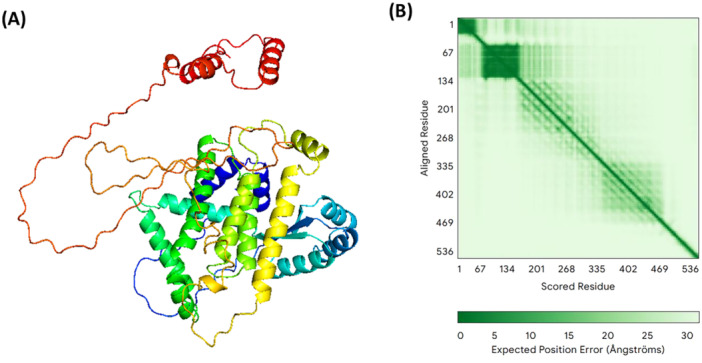
Tertiary structure of the vaccine construct. (A) Vaccine 3D model predicted by AlphaFold. (B) pLDDT value predicted by AlphaFold.

The three‐dimensional structure of the chimeric vaccine construct has been predicted using the AlphaFold AI model. The model of highest accuracy, rank 1, has been considered as it has the highest average pLDDT value, as well as a low Predicted Aligned Error (PAE) value. The structure model has a well‐folded N‐terminal domain represented by the L7/L12 adjuvant, rendering the native α‐helix scaffold unaltered. The adjuvant sequence is distinctly separated from the linear multi‐epitope part by a stiff EAAAK linker, thus not overlapping with the presentation of the epitopes. Figure 7 (A‐B) below represents the 3D structure of the vaccine construct predicted by AlphaFold.

### 3.8 Molecular Docking With TLR4 Receptor

The interaction between the designed multi‐epitope vaccine and human TLR4‐MD2 immune receptor complex was explored through the program ClusPro 2.0. A run of docking generates several possible conformations, sorted in order of RMSD and binding energy patterns into clusters. Table 4 represents the docking energies of the top 10 docking complexes.

**Table 4:** The docking energies of the top 10 docking complexes.

Cluster	Members	Representative weighted score	Lowest energy score
0	73	−1007.8	−1056.3
1	38	−956.6	−1193.0
2	31	−927.1	−1045.8
3	30	−1020.1	−1020.1
4	24	−969.7	−969.7
5	23	−1050.3	−1074.8
6	21	−855.0	−1041.0
7	20	−1002.3	−1111.1
8	20	−855.8	−977.7
9	20	−866.0	−1007.9

**Figure 8:**
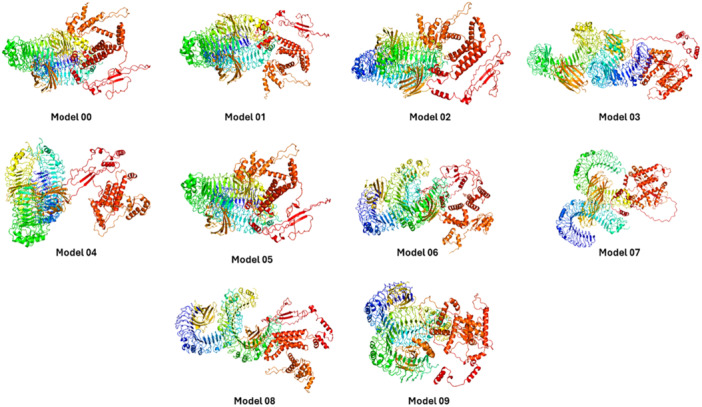
The visualization of the top 10 docking complexes generated by ClusPro 2.0 and visualized by PyMol Graphics Visualization System.

Cluster 0 ranked first and was selected for detailed inspection as the most probable binding mode. It comprised the biggest cluster size, comprising 73 members, indicating very strong convergence toward this orientation. The representative derived from this cluster had a weighted center energy of −1007.8 and a lowest energy score of −1056.3, suggesting a favorable docking score within the ClusPro scoring framework. Although clusters 1 and 29 displayed much lower energy minima at −1193.0 and −1252.8, respectively, Cluster 0 was favoured since its high population density is believed to be a more reliable signal of a native‐like binding state in rigid‐body docking. The Figure 8 (Model 1–10) below represents the top 10 docking complexes visualized on PyMOL.

**Figure 9:**
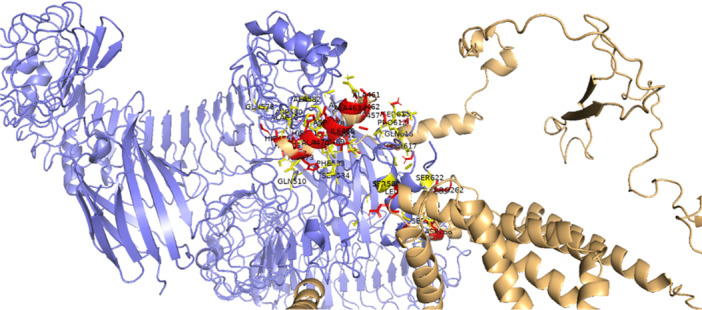
The interacting residues of the vaccine and TLR4 receptor docking complex visualized on the PyMol visualization system.

PyMOL was used to profile the docked complex for mapping of the specific intermolecular contacts. Indeed, the analysis revealed a strong network of hydrogen bonds and hydrophobic interactions at the vaccine–receptor interface, which underlines the vaccine's potential to effectively engage the innate immune system. Figure 9 represents the interacting residues of the best docking complex calculated and visualized in the PyMol Visualization Systems.

### 3.9 Interaction and Stability Analysis

**Figure 10:**
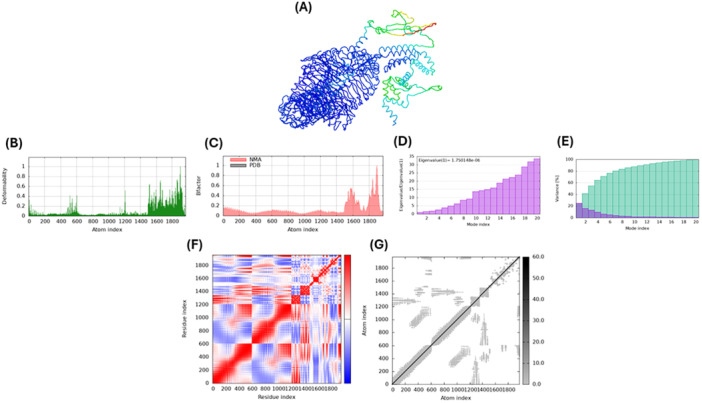
Molecular dynamics simulation and normal mode analysis (NMA) of the vaccine‐TLR4 complex generated by iMODS. (A) NMA mobility analysis showing the 3D structure colored by flexibility (blue: rigid, red: flexible). (B) Deformability plot showing peaks in flexible regions (hinges/linkers). (C) B‐factor values representing atomic fluctuations. (D) Eigenvalue plot indicating the stiffness of the motion (Eigenvalue = 1.750148e‐06). (E) Variance plot showing individual (purple) and cumulative (green) variance explained by the normal modes. (F) Covariance matrix map indicating correlated (red), anti‐correlated (blue), and uncorrelated (white) residue motions. (G) Elastic network model defining the connectivity and stiffness between atom pairs.

The stability of the vaccine‐TLR4 complex was analysed as well as the character of its mobility by using Normal Mode Analysis via the iMODS server (Figure 10A). On inspecting the main chain deformability (Figure 10B), the team observed peaks, especially toward the C‐terminus (atoms 1400–1800), indicating that those areas are more flexible, possibly corresponding to the linker or loop sections that line up for antigen presentation, while the rigid parts correspond to the main sections that maintain a stable fold.

The B‐factor values (Figure 10C), which represent the amplitude of atomic movement, agreed well with the deformability scores, thus validating that the predicted structure has some hard parts and some elastic regions. The eigenvalue of the complex, which correlates with the amount of energy that needs to be invested for the conformation change of the structure, was 1.750148e‐06 (Figure 10D). A smaller eigenvalue suggests that the complex has the ability to easily deform itself in binding reactions.

From the variance analysis (Figure 10E), it can be seen that the first few normal modes encompass the dominant part of the total motion, with the cumulative variance (green bars) approaching saturation, implying that large‐scale motions are largely the result of the action of a few modes with low frequencies. Then, the covariance plot (Figure 10F) revealed the residues that move together or opposite to others: red patches denote residues that move in the same direction, and blue patches, residues that move in opposite directions. The presence of different red patches indicates that the vaccine and the receptor components move largely as rigid units. Finally, the elastic network (Figure 10G) focuses attention on how stiff the links are between pairs of atoms. Darker grey points denote stiffer springs, again implying that the docked complex has good structural integrity. Figure 10A–G represents the results of normal mode analysis predicted by iMODS.

### 3.10 Immune Simulation

Simulation for Immune Response based on the C‐ImmSim server suggests that the multi‐epitope vaccine has the ability to induce the generation of both humoral and cellular immune responses in the body. It should be noted that there is an increased population of B‐lymphocytes and T‐helper cells, indicating the establishment of an immune memory response. The pattern of antibodies generated from this vaccine starts with a burst of IgM production, followed by an increased level of IgG1 and IgG2, which means that the vaccine has the capacity to eliminate antigens.

**Figure 11:**
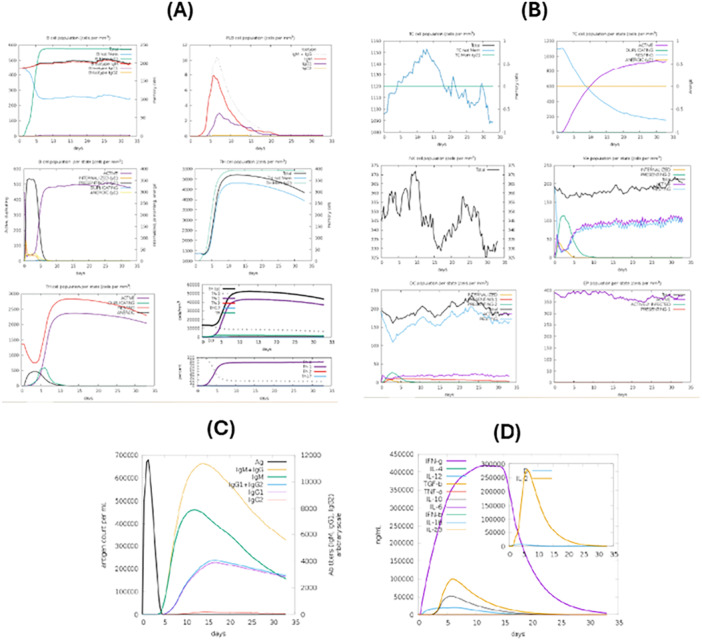
*In silico* immune simulation analysis of the multi‐epitope vaccine generated by C‐ImmSim. (A) Cell population dynamics showing the expansion and memory formation of B‐cells, T‐helper (Th) cells, and Cytotoxic T (Tc) cells. (B) Cytotoxic T‐cell population per state (active, resting, anergic). (C) Immunoglobulin production profile displaying the kinetics of IgM, IgG1, and IgG2 antibodies alongside antigen clearance (black line). (D) Cytokine and interleukin concentration plot highlighting the induction of high levels of IFN‐$\gamma$ and IL‐2.

The plots from the dynamic simulations of Figure 11A–D summarize the time course of the immune response. Cell population data are shown in Figures 11A,B: prominent expansions in B‐cells and Th cells are indicative of immunological memory. This follows logically from the immunoglobulin profile described in Figure 11C, where a rapid IgM response is followed by a more sustained IgG1 and IgG2 antibody titer increase, consistent with efficient antigen clearance. Correspondingly, there is also a high IFN‐γ and IL‐2 cytokine profile from Figure 11D, indicating a strong Th1‐skewed response, which would favour defence against intracellular pathogens.

### 3.11 Codon Optimization and In Silico Cloning

**Figure 12:**
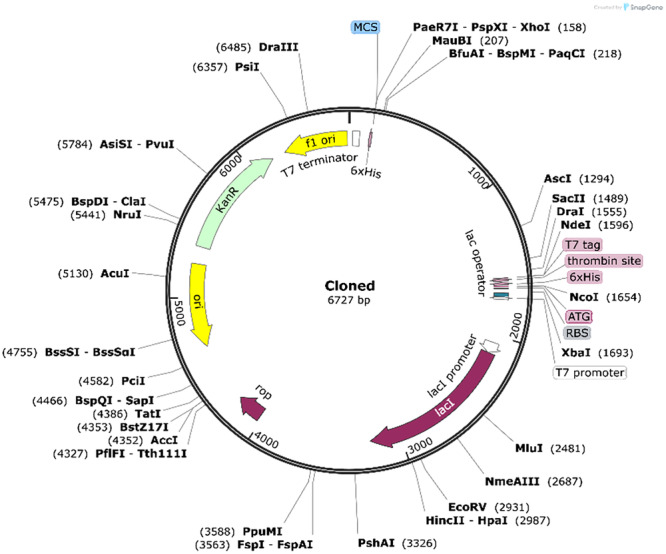
*In silico* cloning of the optimized multi‐epitope vaccine gene into the pET‐28a(+) expression vector. The map illustrates the Cloned insert (6727 bp) relative to key vector elements such as the T7 promoter, lac operator, antibiotic resistance gene (KanR), and origin of replication (f1 ori and ori). The vaccine gene is flanked by restriction sites and contains a C‐terminal 6xHis tag for purification.

The multi‐epitope vaccine, which is made up of 476 amino acids, was reverse translated to create a 1428 base pair nucleotide code. An optimization technique called codon optimization was used to improve levels of gene expression. Codon optimization increased the gene's CAI score substantially. Its original score was 0.34. After optimization, it increased to 0.96. There was also an increase in the GC content of the code. This increased to 70.31% compared to the original 59.59% of the original DNA code. After optimization, the gene was virtually cloned into a pET‐28a+ gene expression system. Its plasmid map measured a total of 6,727 bp. This includes the vaccine gene, a KanR gene as a positive selection marker, as well as a C‐terminal His Tag. Figure 12 represents the cloned vaccine construct in the pET‐28(+) vector system.

## 4 Discussion

The increasing incidence of multidrug‐resistant *Enterobacter cloacae* highlights the need for alternative therapeutic and preventive measures other than the conventional antibiotics. Such a susceptibility to obtain and spread resistance determinants has resulted in this pathogen being an important cause of nosocomial infections, with few treatment options. Vaccination against MDR *E. cloacae* is a prophylactic and sustainable measure of reducing the infection rates, restricting the use of antibiotics, and eventually reducing the appearance of resistance. In this regard, it is quite rational to choose the Outer Membrane Protein A (OmpA) as the vaccine target. OmpA is a highly conserved, surface‐exposed protein that is essential for bacterial adhesion, invasion, immune evasion, and biofilm formation, ensuring the organism's pathogenicity. Its high antigenicity (Jeannin et al. 2022; Ioannou et al. 2022), predicted non‐allergenicity and lack of significant human homology support its suitability as a potential vaccine candidate. In addition, structural characterization of OmpA showed the presence of a classic bimodular structural design, comprising an N‐terminal β‐barrel domain and C‐terminal α‐helical domain. N‐terminal 8‐barrel domain and a C‐terminal 8 ‐helical domain, which contributes to its stability and accessibility to immune surveillance. All these attributes help in enhancing the reason why OmpA is a central antigenic candidate in the construction of vaccines (Yadava et al. 2025; Subedi et al. 2025).

Further immunogenic potential of the target protein and the vaccine construct is supported by the structural properties of the target protein and the vaccine construct. The PSIPRED and AlphaFold studies revealed well‐defined secondary and tertiary structures typical of functional outer membrane proteins, indicating that the antigen can maintain the conformational integrity required to appropriately demonstrate epitopes. The vaccine construct's application of flexible coil sections, primarily through linker sequences, is considered an advantage since it promotes epitope processing and access to immune cells (Bolourchi et al. 2022). The proper prediction and selection of B‐cell, MHC class I, and MHC class II peptides also made sure that peptides with the potential to contribute to humoral and cellular immune responses. The high binding affinity to various HLA alleles and low IC 50 s of these epitopes affirm the high immunogenicity of these epitopes. In addition, the combination of seven B‐cell epitopes, seven epitopes of MHC class I, and four epitopes of MHC class II into a single chimeric construct increases the diversity of immune response and minimizes the chances of immune escape. This multi‐epitope approach enables the vaccine to induce production of antibodies, cytotoxic T‐cells, and helper T‐cells together, which is necessary in effective immune protection against invasive Gram‐negative bacterial infections (Goutam and Shamsuzzaman 2023).

The analysis of the IEDB population coverage indicated that the global coverage reached 99.92 which is remarkable and indicates that the vaccine can be effective among different ethnic and genetic groups (Halder et al. 2024). The large values of the pc90 and hit also indicate that vaccinated persons will tend to identify more epitope‐HLA combinations, which will increase the strength of immunity. Moreover, the molecular docking experiments suggesting a favorable interaction between the vaccine construct and the TLR4‐MD2 complex. This interaction indicates that the vaccine can be successfully used to stimulate the innate immune system, which stimulates antigen presentation and adaptive immune activation, as TLR4 has a crucial role in triggering innate immune responses to Gram‐negative bacteria. The large size of the cluster and good binding energies obtained using ClusPro represent a favorable docking profile within the ClusPro scoring framework. Normal mode analysis also supported the fact that the vaccine −TLR4 complex has an optimal compromise stability‐flexibility balance. This implies that the complex is able to undergo the required conformational changes during immune signaling and retain its overall structural integrity due to the acceptable flexibility and collective motion of the docked complex (Yaikhan et al. 2025).

Simulation outcomes of the immune system show strong evidence of immunogenicity of the proposed vaccine. The rise in numbers in B‐cells, T‐helper, and cytotoxic T‐cells indicates that humoral and cellular immunity have been successfully elicited, and immunological memory has been created. The usual and ideal vaccine‐induced immune profile is demonstrated by the characteristic shift from an initial IgM response to a long‐term IgG1 and IgG2 response. Th1‐biased response, indicated by elevated IFN‐g and IL‐2 is of importance especially when dealing with intracellular infections associated with bacteria. Moreover, codon optimization and *in silico* cloning indicated that the expression of the vaccine construct is possible in an experiment. The large value of CAI and optimal GC content shows high compatibility with the E. coli expression system, and the cloning into the pET‐28a(+) vector will guarantee effective production and purification. Although bacterial OmpA has been reported to engage TLR2, this study prioritized TLR4 for molecular docking analysis due to its pivotal role in initiating the MyD88‐dependent and TRIF‐dependent signaling pathways. These pathways are essential for the robust production of Type I interferons and the Th1‐skewed response observed in our simulations, which are vital for the effective clearance of Enterobacteriaceae. Furthermore, many vaccine adjuvants and multi‐epitope constructs are designed to target TLR4 to maximize the maturation of dendritic cells and the subsequent activation of the adaptive immune system (Reed et al. 2016).

Though these findings are promising, the study is still limited by the use of computational predictions. The safety, stability, and protective efficacy should be verified with the help of experimental validation in the form of in vitro expression and purification, immunogenicity studies, and in vivo animal models. Future research must also conduct research on the formulation strategies, adjuvant optimization, and delivery systems to improve the performance of the vaccines. Although the epitope selection prioritized high‐affinity binding and host non‐homology, explicit strain‐wide conservancy and cross‐reactivity mapping remain vital considerations for ensuring broad‐spectrum efficacy against diverse clinical isolates in future in vitro validations. While the 476‐residue length of the construct presents potential challenges for solubility and stability, these factors were mitigated through optimized linker selection and physicochemical screening, with the inherent limitations of large‐scale antigen design.

## 5 Conclusion

In conclusion, this study suggests a design of a multi‐epitope vaccine against multidrug‐resistant *Enterobacter cloacae*, and OmpA is a highly appropriate antigen since it is conserved, exposed on the surface, and is also critically important to bacterial pathogenicity. The integrated application of Immunoinformatics and epitope prediction, structural modeling, docking, population coverage, immune simulations, and codon optimization is a good indication that the developed vaccine can potentially result in a balanced immune response. Their ability to induce predicted activation of both innate and adaptive immune responses, especially in the context of interaction with the TLR4 receptor and the formation of effective humoral and cellular responses, makes it a promising preventive measure against MDR infections. These results, however, are obtained by means of computational studies that should be checked by experiments. Further efforts in the future should aim at the laboratory expression and purification of the vaccine construct, and then *in vitro* and *in vivo* testing to determine its immunogenicity, safety, and protective effects. To improve their efficacy, further optimization of the vaccine formulation, adjuvant selection, and delivery systems will also be required. Provided its successful validation, the vaccine candidate will be a significant milestone in the reduction of the burden of MDR E. *cloacae* infections and the reduction of dependence on antibiotics.

## Author Contributions

**Maha A. Aljumaa:** project administration, resources, methodology, visualization, validation. **Fakhria A. Al‐Joufi:** investigation, writing – original draft, formal analysis. **Ghulam Nabi:** writing – review and editing; investigation, project administration, data curation, software. **Mengue Ngadena Yolande Sandrine:** conceptualization, funding acquisition, supervision.

## Ethics Statement

The authors have nothing to report.

## Consent

The authors have nothing to report.

## Conflicts of Interest

The authors declare no conflicts of interest.

## Data Availability

The data that supports the findings of this study are available in the supporting material of this article. All the data generated in this research work has been included in the manuscript.
